# Effect of Semaglutide on Physical Function, Body Composition, and Biomarkers of Aging in Older Adults With Overweight and Insulin Resistance: Protocol for an Open-Labeled Randomized Controlled Trial

**DOI:** 10.2196/62667

**Published:** 2024-09-13

**Authors:** Tiffany M Cortes, Libia Vasquez, Monica C Serra, Ronna Robbins, Allison Stepanenko, Kevin Brown, Hannah Barrus, Annalisa Campos, Sara E Espinoza, Nicolas Musi

**Affiliations:** 1 Division of Endocrinology Department of Medicine University of Texas Health Science Center San Antonio San Antonio, TX United States; 2 Sam & Ann Barshop Institute for Longevity & Aging Studies University of Texas Health Science Center San Antonio San Antonio, TX United States; 3 San Antonio Geriatric Research, Education, and Clinical Center South Texas Veterans Health Care System San Antonio, TX United States; 4 Texas Diabetes Institute University Health System San Antonio, TX United States; 5 Division of Geriatrics, Gerontology & Palliative Medicine Department of Medicine University of Texas Health Science San Antonio San Antonio, TX United States; 6 Department of Nutrition and Food Science Texas Woman’s University Denton, TX United States; 7 Center for Translational Geroscience Department of Medicine Cedars-Sinai Medical Center Los Angeles, CA United States; 8 Diabetes and Aging Center Department of Medicine Cedars-Sinai Medical Center Los Angeles, CA United States

**Keywords:** glucagon-like peptide, lean body mass, physical function, biomarkers of aging, semaglutide

## Abstract

**Background:**

Older adults with type 2 diabetes mellitus (T2DM) or prediabetes are at increased risk of adverse changes in body composition, physical function, and aging-related biomarkers compared to those with normal glucose tolerance. Semaglutide is a glucagon-like peptide 1 receptor agonist that has been approved for T2DM and chronic weight management. Although semaglutide is effective for weight loss and T2DM management, its effects on lean body mass, physical function, and biomarkers of aging are understudied in older adults.

**Objective:**

This study aims to compare the effects of lifestyle counseling with and that without semaglutide on body composition, physical function, and biomarkers of aging in older adults.

**Methods:**

This is an open-label randomized controlled trial. A total of 20 adults (aged 65 years and older) with elevated BMI (27-40 kg/m^2^) and prediabetes or well-controlled T2DM (hemoglobin A_1c_ 5.7%-7.5%) are recruited, stratified by sex, and randomized 1:1 to one of 2 groups (semaglutide plus lifestyle counseling vs lifestyle counseling alone) and followed up for 5 months. Those in the semaglutide group are titrated to 1 mg weekly, as tolerated, for 12 weeks. Lifestyle counseling is given by registered dietitians and based on the Diabetes Prevention Program Lifestyle Change Program. Our primary outcomes include changes in lean mass, physical function, and biomarkers of aging. Body composition is measured by dual-energy x-ray absorptiometry and includes total fat mass and lean mass. Physical function is measured by 6-minute walk distance, grip strength, and short physical performance battery. Biomarkers of aging are measured in blood, skeletal muscle, and abdominal adipose tissue to include C-reactive protein, interleukin-6, tumor necrosis factors α, and β galactosidase staining.

**Results:**

The study was funded in December 2021 with a projected data collection period from spring 2023 through summer 2024.

**Conclusions:**

Despite the elevated risk of adverse changes in body composition, physical function, and biomarkers of aging among older adults with glucose intolerance and elevated adiposity, the benefits and risks of commonly prescribed antihyperglycemic or weight loss medications such as semaglutide are understudied. This study aims to fill this knowledge gap to inform clinicians about the potential for additional clinically meaningful, nonglycemic effects of semaglutide.

**Trial Registration:**

ClinicalTrials.gov NCT05786521; https://clinicaltrials.gov/study/NCT05786521

**International Registered Report Identifier (IRRID):**

DERR1-10.2196/62667

## Introduction

### Background

Glucose intolerance affects many older adults in the United States with 49% having prediabetes and an additional 29% having type 2 diabetes mellitus (T2DM) [1]. The mechanisms that lead to age-related glucose intolerance and T2DM are unknown, but age-dependent decreases in β-cell function and insulin sensitivity are thought to be important in the deterioration of glucose homeostasis with age [2]. Unfavorable changes in body composition observed with aging can contribute to insulin resistance, including redistribution of fat from peripheral subcutaneous areas to a central location [3] and loss of lean muscle mass [4]. Overall, changes observed with aging appear to lead to glucose intolerance and T2DM.

Although T2DM complications are primarily thought of as nephropathy, neuropathy, and retinopathy, T2DM is associated with detrimental changes in physical function and body composition, and biomarkers of aging. A recent meta-analysis reports a 50%-80% increased risk of mobility disability; defined as walking speed, chair stand time, and balance test; for those with T2DM compared to people without T2DM [5]. In multiple longitudinal studies, older adults with T2DM have a greater decline in lean mass compared to those without T2DM [6-8]. Additionally, those with prediabetes compared to normoglycemia appear at increased functional risk [9]. These data suggest that insulin resistance, even in the early stages, is associated with adverse changes in body composition and physical function.

Historically, glycemic control has been the focus of T2DM management but the nonglycemic cardiovascular, renal, and weight benefits of some classes of antihyperglycemic agents lead to increased individualization of T2DM therapy regimen based on a patient’s co-morbidities even after glycemic goals are reached. Semaglutide is a glucagon-like peptide 1 (GLP-1) receptor agonist. GLP-1 receptor agonists use the physiologic action of the incretin GLP-1 to increase insulin secretion and suppress glucagon release in response to meals. These drugs reduce hemoglobin A_1c_ (HbA_1c_) by 1.45%-1.55%, depending on dose [10,11]. Semaglutide additionally has nonglycemic benefits, including chronic weight management and reduction of cardiovascular events. A recent meta-analysis determined that semaglutide has an average weight loss of 12% with 54% of participants achieving at least 15% weight loss [12]. Currently, semaglutide is approved by the Food and Drug Administration not only for T2DM management, to decrease cardiovascular events in adults with T2DM, and for chronic weight management for those with a BMI of ≥30 kg/m^2^ or ≥27 kg/m^2^ with at least 1 weight-related comorbidity Therefore, a significant portion of older adults may be prescribed GLP-1 receptor agonists considering that 29% of them have T2DM and 42.8% of them have a BMI of ≥30 kg/m^2^ [1,13].

With significant weight loss resulting from semaglutide use, concomitant lean body mass losses may occur. However, the results on lean mass appear mixed, with most trials indicating that semaglutide results in reductions in total fat mass and lean mass with most of the weight loss resulting from fat mass loss [14-17], while others revealed that lean body mass remained stable [18-20]. The effect of semaglutide on physical function has been infrequently studied in older adults. One study found the preservation of grip strength [14] but have not evaluated other validated measures of function in older adults such as short physical performance battery and 6-minute walk distance. Additional measures of muscle quality such as histology and mitochondrial respiration have not been completed in humans. Of note, these trials have an average studied population younger than 65 years, limiting the ability to extrapolate the results to older adults. These data suggest a gap in the literature related to the effects of semaglutide on body composition and physical function in older adults.

Since semaglutide has positive changes in glycemic control, cardiovascular system, and weight management; there are possible changes to the aging process as well. Although there is no standard set of biomarkers to obtain for evaluation of aging, systemic interleukin (IL)-6, tumor necrosis factors α (TNFα), C-reactive protein (CRP), and GDF15 are proposed biomarkers that are used in geroscience guided clinical trials [21]. A process that contributes to aging is cellular senescence, which is a stable cell cycle arrest triggered in normal cells that can lead to impaired tissue regeneration and chronic age-associated disease [22]. Cellular senescence can be detected by staining tissues for senescence-associated β-galactosidase and senescence-associated secretory phenotype [22]. Although semaglutide has not been widely studied, the class of GLP-1 receptor agonists shows evidence of improvement in CRP and TNFα [23] with 1 study indicating that CRP has a significant decline following 68 weeks of treatment with semaglutide [24]. Therefore, this underscores the necessity for further research in this field.

### Objectives

This study aims to (1) ascertain the potential ramifications of semaglutide on the lean mass profiles and physical functionality of individuals with prediabetes or T2DM and (2) to assess the impact of semaglutide on molecular biomarkers of aging in blood, adipose, and skeletal muscle tissues. We hypothesize that semaglutide will induce favorable alterations in body composition, particularly for lean and adipose body mass quantified via dual-energy x-ray absorptiometry, and improve physical function, as assessed by metrics such as the short physical performance battery, handgrip strength, and walking speed. Additionally, clinical changes are associated with positive changes in systemic and tissue-specific biomarkers of aging.

## Methods

### Trial Design

This is a randomized, open-label pilot study. The institutional review board is notified of any deviations from the approved protocol or breaches of confidentiality.

As shown in Table 1, this study involves 8 visits, which include screening and baseline assessments (visit 1 and visit 2), randomization (visit 2), intervention follow-up (visits 3-5), end-of-study assessments (visits 6a and 6b), and a postintervention telemedicine follow-up visit (visit 7). The intervention phase occurs over 20 weeks. Participants are contacted before in-person visits to ensure they are able to attend or reschedule if needed. All in-person visits are conducted at the Sam and Ann Barshop Institute for Longevity and Aging Studies, University of Texas Health Science Center at San Antonio.

**Table 1:** Schedule of events.

	Screening	Intervention start, T=0 week	T + 3 weeks (±7 days)	T + 7 weeks (±7 days)	T + 11 weeks (±7 days)	T + 20 weeks (±7 days)	T + 20 weeks (±7 days)	T + 21 weeks (± 7 days)
Visit	V1	V2	V3	V4	V5	V6a^a^	V6b^a^	V7
Medical history and physical exam	X					X		
Vitals, anthropometric measurements	X	X	X	X	X	X	X	
Clinical labs	X					X		
Randomization		X						
DXA^b^ scan	X					X		
ECG^c^	X							
SPPB^d^, grip strength, 6-minute walk	X					X		
Surveys (SF-12^e^, CNAQ^f^)	X					X		
Blood, muscle, and adipose biopsy		X					X	
Start of semaglutide for treatment group		X						
Semaglutide dispensed to treatment group		X	X	X	X			
Lifestyle education		X	X	X	X			
Assessment of adverse events		X	X	X	X	X	X	X

^a^Visits 6a and 6b are the end-of-study visits but are split into 2 visits.

^b^DXA: dual-energy x-ray absorptiometry.

^c^ECG: electrocardiogram.

^d^SPPB: short physical performance battery.

^e^SF-12: Short-Form 12.

^f^CNAQ: The Council on Nutrition Appetite Questionnaire.

### Participants and Inclusion and Exclusion Criteria

The study is recruiting 20 older adults from San Antonio, Texas. Overall, for this pilot, our population is generally healthy older adults who are eligible to receive semaglutide for either indication of T2DM or chronic weight management. The inclusion criteria are as follows: (1) men and postmenopausal women; (2) aged 65 years and older; (3) BMI: 27-40 kg/m^2^; (4) HbA_1c_ 5.7%-7.5% or fasting blood glucose greater than 100 mg/dL; (5) community-dwelling; (6) willingness and ability to comply with protocol requirements. The exclusion criteria are as follows: (1) glomerular filtration rate≤29 mL/min/1.73 m^2^ [25]; (2) hematocrit≤33%; (3) thyroid stimulating hormone>7 mIU/L with abnormal free thyroxine; (5) use of medications known to lower blood glucose besides metformin; (6) history of cardiovascular events; (7) systolic blood pressure≥170 mm Hg or diastolic blood pressure≥95 mm Hg; (8) active inflammatory, autoimmune, infectious, hepatic, gastrointestinal, malignant, or uncontrolled psychiatric disease; (9) ≥5% change in weight during the 3-month period prior to screening; (10) personal or family history of medullary thyroid cancer [26]; (11) personal or family history of multiple endocrine neoplasia syndrome type 2 [26]; (12) previous history of pancreatitis [26]; (13) electrocardiogram with QTc prolongation (QTc>470 ms in men or >480 ms in women) or evidence of ischemic changes; (14) personal history of diabetic retinopathy [26]; (15) allergy to semaglutide; and (16) type 1 diabetes or other insulin dependent diabetes. Individuals who initially fail their initial screening are eligible to be rescreened 12 weeks after their initial screening.

### Recruitment

Recruitment methods include electronic medical records and Barshop Call Center Registry queries, community engagement activities, and web-based advertisements (including social media). Potential participants undergo a prescreening questionnaire to ensure they meet the inclusion and exclusion criteria for enrollment. Those who qualify from the prescreening questions are scheduled for a full in-clinic screening evaluation and sent an advance copy of the consent form.

### Screening

We conduct an in-clinic evaluation consisting of medical and social history, current medications, vital signs, anthropometric measurements, and physical examination. Additionally, a fasting blood profile is collected to assess a complete metabolic panel, complete blood count, HbA_1c_, thyroid stimulating hormone, and coagulation panel. Body composition analysis, evaluation of physical function, and completion of patient-reported measures, as detailed below, also are carried out during this visit.

### Baseline and Postintervention Assessment Measures

Assessments are performed at baseline (screening or visit 1) and the end of the study (visits 6a and 6b) for both groups (Textbox 1).

**Study assessment measures.**

**Laboratory tests**

Clinical labs: After fasting overnight, whole blood is collected for the assessment of complete blood count, comprehensive metabolic panel, hemoglobin A_1c_, lipid profile, thyroid stimulating hormone, prothrombin time, and partial thromboplastin time.

Research laboratories: After fasting overnight, a credentialed research team member obtains blood, skeletal muscle, and abdominal adipose samples. Blood is drawn by staff well-versed in phlebotomy to reduce the number of attempts needed for venipuncture. Vastus lateralis muscle biopsy are obtained with a Bergstrom needle after location anesthesia is achieved. Assessments of inflammation and cellular senescence in muscle, adipose tissue, and blood samples are conducted within the clinical research unit. In adipose tissue, we perform staining for β-galactosidase [27]. For muscle tissue, histological processing and measurement of mitochondrial respiration are carried out immediately, followed by freezing additional tissue for the analysis of interleukin (IL)-6, tumor necrosis factor α (TNFα), TNFα receptors 1 and 2, p12, and p16 concentrations. We also investigate changes in SIRT1 activity within muscle tissue. Plasma samples obtained from whole blood are used to measure concentrations of IL-6, TNFα, and C-reactive protein.

**Anthropometric measurements**

BMI: Weight is measured on a standard, calibrated scale at baseline and throughout the study without shoes and with light clothing. Standing height (cm) is measured using a stadiometer at screening. BMI is calculated as weight (kg)/ height (m^2^).

Waist circumference: Waist circumference is measured with a tape measure at the level of the iliac crest.

Body composition: dual-energy x-ray absorptiometry scans are used to measure whole-body adipose and lean mass and total bone mineral density.

**Physical function**

Short physical performance battery: A standardized and validated assessment developed by the National Institute on Aging for use in the Established Population of the Epidemiologic Studies of the Elderly, which is used to evaluate the physical function of older adults independently from their self-reported abilities [28]. The tests fall under 3 categories: balance, gait speed, and chair stand tests. The entire battery of tests can be finished within 10-15 minutes. The administrator begins by demonstrating all the activities to the participant. The balance assessment comprises side-by-side, semitandem, and tandem stands. The gait speed test involves walking along a 4-meter path at their usual pace, repeated twice. Finally, participants are assessed on their ability to rise from a chair without arm assistance, followed by completing 5 timed chair rises. The maximum score is 12. We will classify a score <10 as an increased risk of all-cause mortality [29].

Grip strength: Hand grip strength of both the dominant and nondominant arms ware assessed using a handheld dynamometer [30]. The participant’s hand grip strength is assessed and the average grip strength from the 3 trials recorded. This test takes less than 5 minutes. Grip strength for women <16 kg and men >26 kg will be classified as at risk [31].

6-minute walk: Participants are asked to walk as quickly as they can, for as many times as they can over a 40-foot track for a total of 6 minutes. The total distance walked is obtained [32]. Values will be compared to normative values for healthy older adults: 367 m for women and 400 m for men [33].

**Patient-reported outcomes**

The Council on Nutrition appetite questionnaire (CNAQ): Validated as an evaluation of appetite in older adults to predict anorexia before weight loss [34]. The CNAQ consists of 8 items within a single domain, with responses rated on a 5-point scale. The total score is calculated by summing the scores of all 8 items, ranging from 8=indicating the poorest appetite to 40=indicating the best appetite.

The Short- Form 12: A validated tool for assessing health-related quality-of-life in older adults. A higher score indicated better health with a score of 50 indicating the average score in the general US population [35].

### Intervention

#### Randomization

Eligible participants are randomized to either the lifestyle counseling group (control) or the semaglutide with lifestyle counseling group (1:1) after their blood draw and muscle and adipose biopsies during visit 2. Randomization is performed by the principal investigator at the end of the visit 2 through tables that were stratified by sex in sequential order at the time of randomization. A total of 10 participants are to be enrolled in each group, with 20 overall enrolled. Due to cost restrictions, placebos are not used, and the principal investigator and the participants are unblinded.

#### Lifestyle Counseling

All participants meet with a registered dietitian for an hour-long diet, exercise, and behavior modification sessions at visits 2-5. When in-person classes are not feasible, web-based sessions are made available. Behavior modification is based on the Diabetes Prevention Program Lifestyle Change Program [36]. Aligned with the Diabetes Prevention Program participants receive instructions for changing their dietary composition based on the US Department of Agriculture’s MyPlate guidelines, which focus on fruit, vegetables, low-fat dairy, and whole grains. MyPlate reinforces small feasible and affordable changes that result in sustainable eating patterns that promote good health [37].

Participants are given detailed written and verbal instructions to achieve these guidelines. During these sessions, education materials are provided on budget-friendly nutrition for optimal aging: nutrition basics (ie, healthy dietary patterns, calorie balance, food labels, and hydration), recipe modification, and mindful eating. In addition, based on the American College of Sports Medicine physical activity recommendations (duration, intensity, and mode) participants are encouraged to perform moderate-intensity physical activity at least 150 minutes per week [38]. Physical activity instruction also focuses on how to safely exercise in their home environments. We also cover topics based on participants’ questions, which we anticipate may include managing common comorbidities (ie, sleep behaviors, preventing and managing diabetes, avoiding excessive sitting, and managing stress), and available community resources. At each follow-up lifestyle session, weight is obtained, and previously made goals are assessed to determine success or additional modifications required to achieve goals.

#### Semaglutide

Participants are given verbal and written instructions on appropriate preparation and subcutaneous injection of semaglutide are self-administered once a week. At visit 2, a semaglutide pen that administers 0.25-0.5–mg dose is provided and participants are monitored as they self-administer their first dose of 0.25 mg. They continue semaglutide 0.25 mg for additional 3 weeks, then increase as tolerated to 0.5 mg on visit 3 for 4 weeks, and 1 mg once a week on visit 4 for 4 weeks and continue 1.0 mg on visit 5 for an additional 8 weeks. The titration planned is as follows: complete 4 weeks of 0.25 mg, 4 weeks of 0.5 mg, and 12 weeks of 1.0 mg. If titration is not tolerated, then the participant is kept on the tolerated dose for a longer period with a retry of the higher dose at the next visit.

At each visit, participants are given a semaglutide pen, along with a schedule for the date of the next planned self-administered injection. Participants are asked to bring their study medication to every visit to determine if the appropriate weekly dose has been administered. Participants are withdrawn from the study for noncompliance with semaglutide injections or inability to increase the weekly dose.

### Statistical Analysis

The primary outcomes include changes in lean mass, 6-minute walk distance, grip strength, and short physical performance battery after the 20-week intervention between the semaglutide and lifestyle compared to lifestyle alone groups. We use intention-to-treat principles per the Consolidated Standard of Reporting Trials for analysis [39]. Experimental results are expressed as means (SEM). Comparisons of means between groups are performed by analysis of variance for repeated measures and general linear model are used to add covariates. Associations, within a group, between aging-related biomarkers versus body composition and physical function outcomes are determined by Pearson correlation. For tests of correlation coefficients between groups, we use Fisher *Z* transformation. Multiple regression analysis was used to determine the relationship between aging-related biomarkers and body composition and physical function outcomes. Covariates include age, sex, ethnicity, and BMI. Alpha equal to .05 is used to determine significance. We plan for 20 participants, 10 per group, to accomplish this pilot study within the funding mechanism timeframe. The data generated through this study are used to guide sample size and power calculations for further studies. The Biostatistics Core of the San Antonio Claude D Pepper Older Americans Independence Center is assisting with statistical analyses. Planned software analysis program is SAS (version 9.4; SAS Institute).

### Monitoring Adverse Events

Adverse events are assessed during set interactions with the participants. This includes in-person visits at the research facility and a telemedicine encounter during visit 7. They will also be provided with information on how to contact the research team if they have concerns about medication adverse reactions between visits. All adverse events are logged and reported to the institutional review board. Additionally, adverse events are reviewed by a medical doctor to determine the severity, the event’s relationship to the study intervention, and what action should be taken.

### Data Management

Each participant is assigned a study code not associated with their name. Data are saved and stored using REDCap (Research Electronic Data Capture; Vanderbilt University) electronic data management system, which is a secure, password-protected, local area network hosted at the University of Texas Health Science Center at San Antonio. Participants’ charts, which will include all the visits’ documents, are kept inside a locked cabinet located in the clinical research space.

### Ethical Considerations

The study is approved by the University of Texas Health Science Center San Antonio institutional review board (20220256HU) in accordance with the ethical standards of the responsible committees on humane experimentation and the Declaration of Helsinki. Eligible participants sign the informed consent after a research staff member has explained the study and answered all questions. Participants may choose to leave or opt out of the study at any time without penalty. Participants receive compensation for their time up to US $425 through a ClinCard if all visits are completed. Privacy and confidentiality are maintained by having only authorized research staff accessing patient health information, keeping participant files in a locked cabinet, and providing access to REDCap to the minimum authorized research staff. The study is registered with ClinicalTrials.gov (NCT05786521).

## Results

The study was funded in December 2021, with a projected data collection period lasting from Spring 2023 through Summer 2024. Data analysis and results are aimed to be published in late 2024.

## Discussion

### Summary

The purpose of this study is to investigate the effect of semaglutide in addition to lifestyle counseling compared to lifestyle alone on body composition, physical function, and systemic and local biomarkers of aging. We hypothesize that semaglutide therapy will improve body composition and physical function and that these clinical improvements are supported by positive changes to biomarkers of aging.

GLP-1 receptor agonists are a relatively novel group of antihyperglycemic agents that are prescribed in increasing frequency [40]. Thus far, glycemic control, cardiovascular benefits, and whole-body weight reduction have been observed in people treated with semaglutide [10,11,41]. The majority of studies on GLP-1 receptor agonist use suggest a loss of lean mass, though this is not universally observed [18,19]. With the popularity of these medications, the additional effects on health parameters especially important to older adults such as function and lean body mass need to be thoroughly evaluated.

Although there have been few studies examining the effect of semaglutide on measures of health span in older adults, several preclinical studies have been conducted. Semaglutide treatment in obese mice resulted in an increase of type I/II muscle fibers, muscle fiber density, mitochondrial mass, and decline of TNFα, IL6, and IL-1β compared to untreated obese mice, resulting in the semaglutide-treated mice having a muscle quality similar to the nonobese control group [42]. In a mouse model of sarcopenic obesity, where mice were fed a high-fat diet, treatment with semaglutide led to enhancements in muscle strength, muscle fiber characteristics, and markers of muscle atrophy when compared to mice that did not receive treatment. [43]. The improvements in muscle quality and strength seen with semaglutide may be through the SIRT1 pathway, which is a key regulator of skeletal muscle biology and function. SIRT1 has also been associated with the ability of GLP-1 receptor agonists to improve hepatic steatosis and skeletal muscle insulin resistance [44,45]. This study intends to add to this mechanistic research by providing an insight into the effects of semaglutide on biomarkers of aging. These findings may be especially relevant to older adults as clinical targets for future evaluation and potential intervention.

### Limitations

Although this is a randomized controlled trial, there are potential limitations. As this is an open-label trial, the lack of blinding can lead to ascertainment bias [46]. The small sample size for this pilot may lead to a type II error. Given the 5-month duration of the trial, it is possible that maximum weight loss may not be attained. Consequently, we lack a comprehensive understanding of the full impact of this class of medication under conditions of maximum weight loss.

### Conclusions

Based upon the known glycemic, cardiovascular, and body weight loss effects of GLP-1 receptor agonists in the treatment of T2DM and chronic weight management, we are conducting a clinical trial to expand this work by examining the effects of semaglutide on body composition, physical function and biomarkers of aging in older adults with prediabetes and well-controlled T2DM. This study fills a significant gap by focusing on the effect of frequently used medication in older adults and infrequently studied outcomes of clinical significance in this population. The findings will serve as valuable insights for future research endeavors, potentially shaping the treatment approaches using GLP-1 receptor agonists among older adults.

## Appendix

Multimedia Appendix 1 Peer review report from San Antonio Older Adult Independence Center .

## Abbreviations

CRP: C-reactive protein
GLP-1: glucagon-like peptide 1
HbA1c: hemoglobin A1c
IL: interleukin
REDCap: Research Electronic Data Capture
T2DM: type 2 diabetes mellitus
TNFα: tumor necrosis factors α

## Data Availability

Data sharing is not applicable to this article as no data sets were generated or analyzed for this article. The data sets analyzed during this study will be available from the corresponding author on reasonable request.
