# Supplementary material for: Effect of Semaglutide on Physical Function, Body Composition, and Biomarkers of Aging in Older Adults With Overweight and Insulin Resistance: Protocol for an Open-Labeled Randomized Controlled Trial
Source: JMIR Res Protoc. 2024 Sep 13;13:e62667. doi: 10.2196/62667 (PMC11437224; doi:10.2196/62667)
Supplement: Multimedia Appendix 1 [file resprot_v13i1e62667_app1.pdf]

UT Health San Antonio
The Sam and Ann Barshop Institute for Longevity and Aging Studies

June 11, 2024

Reviews for Tiffany Cortes, MD, Pepper Center RL5 application, 2021

Reviewer 1

Overall Impact Score: 2

Strengths

- This is a RL5 Scholar application from a well-trained physician investigator from Department of Medicine at UTHSA.
- The candidate has access to outstanding intellectual and clinical study expertise.
- In this application, the candidate proposes to define the effect of GLP1 receptor agonist on physical function, body composition, and biomarkers of aging in older adult overweight/obese adults with insulin resistance and to yield new insights into the benefical effect of GLP-1 in obese patients during aging.
- This is an innovative area of understanding the role of GLP-1 in older obese adults with prediabetes and in biomarkers of gaining in the clinical trial.
- If successful, these proposed studies will elucidate a novel mechanism by which GLP-1 benefits key aspects of metabolism and aging-related decline in older, obese patients, thereby providing a potential avenue for treatment obesity during aging.
- The mentor/co-mentor, Dr. Sara Espinoza and Dr. Nicolas Musi, have all necessary resources to support the applicant during a RL5 award.
- The mentoring team and the environment at Department of Medicine/Endocrinology are outstanding.
- In sum, this is a very strong application from a talented physician investigator on an interesting problem with clinical significance, deserving of high enthusiasm.

Weaknesses

- However, a minor weakness, such as the less detained description of research approach in Aim#2 for the relationship between body composition and aging biomarkers, is noted.

1. Is the candidate appropriate for the scholar program (at stage of career to benefit, prior training appropriate and good quality)?
Strengths
Weaknesses

2. Is the proposed research plan appropriate for the Pepper Center; do you have any specific comments on the research (optional)? 2
Strengths
Weaknesses

3. Is the mentor(s) appropriately qualified for the scholar program? 1
Strengths
Weaknesses

THE SAM AND ANN BARSHOP INSTITUTE FOR LONGEVITY AND AGING STUDIES | Mail Code 7755 | 7703 Floyd Curl Drive |
San Antonio, Texas 78229-3900
210.562.6140 | Fax 210.562.6150 | barshopinstitute.uthscsa.edu

4. Is the proposed scholarly activity appropriate (include comments on mentoring team if that is proposed)? 1

Strengths
- The mentoring team and the environment at Department of Medicine/Endocriology are outstanding.

Weaknesses

Reviewer 2

Overall Impact Score: 3

Strengths
- Candidate has strong clinical training in medicine/endocrinology
- Excellent mentors
- Overall training plan (seminars, meetings with mentors; classwork) appropriate

Weaknesses
- Rationale for proposal not very strong/ experimental design does not lead to outcomes readily indicative, if positive, of a direct, unique action of the agonist being tested. Hence, outcome, even if positive may be difficult to publish.

1. Is the candidate appropriate for the scholar program (at stage of career to benefit, prior training appropriate and good quality)? 1

Strengths
- MD UT Health, Fellowship in endocrinology Mayo Clinic
- Recent experience in clinical research at Barshop/VA
- Strong interest in becoming independent clinician scientist

Weaknesses
- None detected

2. Is the proposed research plan appropriate for the Pepper Center; do you have any specific comments on the research (optional)? Yes; and Yes (3)

Strengths
- Comprehensive research plan (e.g., Table 1) to train toward independence and get deeper training in Geoscience.
- Aims will provide broad training in many aspect is of clinical/translational research (e.g. patient recruitment; IRB issues, human study design and implementation; biochemical markers/ assessment.

Weaknesses
- I found the overall training plan weakened by the research plan: specifically, the weak rationale and ultimately, difficultly in interpreting the outcomes – especially in terms of whether the intervention (GLP1 agonist) was specifically/directly improving muscle function or merely acting as any T2D drug might act through improving glucose regulation and insulin action.

- Specifically: the applicant notes that GLP1 agonists have been known to reduce lean body mass! The hypothesis is that it will reduce sarcopenia and increase strength – this was not clearly reconciled.
- Second, the controls seemed inadequate to test for specificity of the intervention's effect. One would think having other T2D drugs such as canagliflozin, would be needed to test for specificity of any benefit due to GLP1 antagonist. At least recognition of this weakness should be part of the proposal.
- Third, the hypothesis that GLP1 agonist treatment will reduce cell senescence and that the reduction will be a likely cause of improved muscle function rests on very tenuous arguments: namely, that T2D is associated with increased SASP and cell senescence and that, since GLP1 agonists can reduce T2D symptomatology it is likely acting through reduction of cell senescence.

3. Is the mentor(s) appropriately qualified for the scholar program? 1

Strengths
- Excellent mentors

Weaknesses
- None

4. Is the proposed scholarly activity appropriate (include comments on mentoring team if that is proposed)? 3

Strengths
- See strengths in "2" above.

Weaknesses
- See weaknesses in "2" above.
